# Editorial for “Novel Diagnostic and Therapeutic Approaches in Temporomandibular Disorders and Myofascial Pain” Special Issue in *Life*

**DOI:** 10.3390/life13102049

**Published:** 2023-10-13

**Authors:** Zuzanna Nowak

**Affiliations:** Department of Temporomandibular Disorders, Medical University of Silesia in Katowice, Traugutta sq. 2, 41-800 Zabrze, Poland; zuzannaewanowak33@gmail.com

In the dynamic and complex field of temporomandibular disorders (TMDs), keeping our knowledge up to date is of great importance. The following Special Issue focuses on novel as well as established therapies [1,2,3,4,5,6], with an additional glimpse at diagnostic improving tools and novel approaches [4,7,8], as well as epidemiological data [9].

Diagnosing and screening for TMDs is enduringly a relevant problem for general practitioners. Many patients must visit several specialists, often from various fields, before finding help. Moxley et al., based on the recent literature, pointed to the possibility of introducing an artificial neural network-based program to aid the diagnostic process [4]. Kreiner et al., who created the program, showed its significant advantage over the diagnostic abilities of inexperienced general dental practitioners, as it has a sensitivity and specificity close to 100% [10]. Another method described the assessment of personal salivary endocannabinoid profiles for patients, which could be useful for screening purposes. Another approach was suggested by Diaz-Saez et al., who attempted to assess the intra- and inter-rater reliability of a novel device to evaluate tongue force [7]. In contrast to the methods described above, it is a highly specialistic tool to aid in the search for possible additional TMD risk factors, such as tongue dysfunction.

The understanding of TMDs gradually evolves toward a more biopsychosocial approach where patients’ pain and disabilities are no longer attributed to strictly mechanical disruptions but may originate, in fact, from more complex causes, and their treatment should be adequate [11,12]. It turns out that conventional treatments like occlusal splint therapy, intramuscular and intracapsular injections, manual therapy, and pharmacotherapy can be greatly improved by supplementing them with elements attributed to lifestyle medicine that increase general well-being. Regulating sleep patterns, breathing, dietary habits, and exercising, as well as learning stress management, benefits patients. Such actions allow proper tissue regeneration and an increased pain tolerance threshold, which creates a proper environment for patients’ recovery at the root of the problem. Following general health improvement, a closer look at the orofacial area and local interventions are still necessary. 

Physiotherapy remains a successful local treatment for muscular TMDs. Vieira et al. showed, in a quality systematic review, the evidence for the effectiveness of manual therapy in managing TMDs [1]. Although this approach is nothing novel, there is ongoing research to develop better protocols, as described by Moxley et al. [4]. Similarly, needling therapies constantly reappear in the literature. In this Special Issue, de Sousa et al. and Blanco-Rueda et al. presented their clinical findings as researchers and clinicians still lack standardized protocols for effective therapies [3,5]. With a growing amount of research, the goal should be to determine indications and contraindications for certain substances, the number of sessions for which they should be administered, and their long term effects. A noticeable trend within the studies points towards the use of more natural compounds like collagen, platelet derived plasma (PRP), and hyaluronic acid, which show great effectiveness and safety in needling therapies. In some cases, a last resort treatment has to be applied when patients are qualified for total joint replacement. The indications for such surgery are very limited; however, its benefits for patients in need are enormous, as shown by Speksnijder et al. in their clinical pilot study [6].

The following Special Issue presents a brief cross section through selected topics related to TMDs, bringing some novel insights as well as contributing to topical domains. It is an interesting read that should inspire further investigation within the complex subject of TMDs and point to directions for further clinical research.
